# Supplementary material for: Emergence of a carbapenem-resistant atypical uropathogenic Escherichia coli clone as an increasing cause of urinary tract infection
Source: Nat Commun. 2025 Sep 2;16:8200. doi: 10.1038/s41467-025-63477-0 (PMC12405470; doi:10.1038/s41467-025-63477-0)
Supplement: Supplementary file 1 — Supplementary Information [file 41467_2025_63477_MOESM1_ESM.pdf]

Supplementary information for

Emergence of a carbapenem-resistant atypical uropathogenic *Escherichia coli* clone as an increasing cause of urinary tract infection

Lachlan L Walker[1,2], Minh-Duy Phan[1,2], Budi Permana[1,2], Zheng Jie Lian[1,2], Nguyen Thi Khanh Nhu[1,2], Thom Cuddihy[1,2], Kate M Peters[1,2], Kay A Ramsay[3], Chelsea Stewart[1,2], Niels Pfennigwerth[4], Timothy J Kidd[5], Patrick N.A Harris[3,5], David L Paterson[6], Matthew J Sweet[1,2], Brian M Forde[1,2,3]*, Mark A Schembri[1,2,7]*

[1]Institute for Molecular Bioscience, The University of Queensland, Brisbane, Queensland, Australia

[2]Australian Infectious Diseases Research Centre, The University of Queensland, Brisbane, Queensland, Australia

[3]UQ Centre for Clinical Research, Faculty of Medicine, The University of Queensland, Brisbane, Queensland, Australia

[4]German National Reference Centre for Multidrug-resistant Gram-negative Bacteria, Department of Medical Microbiology, Ruhr-University Bochum, Universitätsstraße 150, 44801 Bochum, Germany

[5]Pathology Queensland-Central Microbiology Laboratory, Queensland Health, Brisbane, Queensland, Australia

[6]ADVANCE-ID, Saw Swee Hock School of Public Health, National University of Singapore, Singapore, Singapore

[7]School of Chemistry and Molecular Biosciences, The University of Queensland, Brisbane, Queensland, Australia

Short title: Emergence of a carbapenem-resistant atypical UPEC clone

Keywords: uropathogenic *Escherichia coli*, ST167, urinary tract infection, uropathogenic *E. coli*, antibiotic resistance, AMR, capsule

Funding: This work was supported by a National Health and Medical Research Council (NHMRC) grant to MAS, M-DP and NTKN, and a Medical Research Future Fund (MRFF) grant to MAS, M-DP and BMF.

Author contributions: Performed the experiments: LLW, BP, ZJL, NTKN, TC, M-DP, KMP, KAR, CS. Conceived and designed the experiments: LLW, M-DP, PNAH, DLP, MJS, BMF, MAS. Analysed the data: LLW, M-DP, BP, KAR, ZJL, NTKN, BMF, MAS. Supervised aspects of the project: M-DP, NP, TJK, PNAH, DLP, MJS, BMF, MAS. Wrote the manuscript: LLW, M-DP, BMF, MAS. All authors read and approved the final manuscript.

Conflict of interest statement: The authors declare that they have no conflict of interest.

***Corresponding authors:**

Professor Mark Schembri, Institute for Molecular Bioscience, The University of Queensland, Brisbane, Queensland 4072, Australia. email: m.schembri@uq.edu.au

Dr Brian Forde, Institute for Molecular Bioscience, The University of Queensland, Brisbane, Queensland 4072, Australia. email: b.forde@uq.edu.au

Supplementary Table 1. Enterobase prevalence of the 20 most prevalent STs in 2024 through the past 14 years from 2010-2024.

Total Isolates for 20 most Prevalent STs																
**ST**	**Year**															**Total Count**
	2010	2011	2012	2013	2014	2015	2016	2017	2018	2019	2020	2021	2022	2023	2024	
10	189	109	506	293	614	994	855	1141	1559	1980	740	818	870	836	371	11875
38	48	60	186	116	181	198	250	374	420	544	267	204	132	160	49	3189
48	4	5	39	31	58	91	174	130	220	145	87	127	102	105	29	1347
58	135	50	251	84	151	185	218	311	348	392	317	313	291	227	71	3344
69	87	35	169	105	179	258	264	407	400	593	495	301	311	212	81	3897
73	73	70	187	95	118	158	146	249	298	637	682	192	322	125	23	3375
88	28	35	88	42	187	244	63	215	145	131	124	101	97	83	26	1609
95	80	66	206	120	164	210	142	272	213	326	467	192	172	88	19	2737
131	410	399	827	672	1099	1069	1100	1447	1676	2689	1425	961	597	473	136	14980
162	19	9	45	24	86	180	81	185	184	185	160	122	126	165	43	1614
167	11	12	25	22	52	101	98	126	164	256	147	175	167	248	197	1801
297	36	16	41	19	33	61	161	57	94	97	91	141	95	82	31	1055
349	6	4	21	20	69	46	20	68	91	84	57	68	37	71	23	685
361	3	3	7	6	60	50	42	41	55	112	72	75	77	99	72	774
405	19	23	59	45	144	100	152	136	137	229	131	87	80	107	78	1527
410	18	34	70	52	163	190	231	243	212	261	160	189	128	171	118	2240
648	17	23	60	55	87	105	109	174	195	283	121	96	78	80	40	1523
1193	15	11	46	55	87	114	107	185	272	393	341	140	101	107	28	2002
504	0	2	1	5	7	27	17	14	26	21	13	8	37	49	38	265
3580	0	3	19	7	2	3	8	43	67	71	35	67	51	51	23	450
All STs	4252	2978	8558	5655	11119	14236	14397	17825	20278	23480	16759	17234	17238	15935	8408	198352

Supplementary Table 2. Enterobase data showing the number of UTI isolates deposited in Enterobase from 2010-2024 for the five most deposited STs in 2024.

UTI Isolates for five most prevalent STs																
**ST**	**Year**															**Total Count**
	2010	2011	2012	2013	2014	2015	2016	2017	2018	2019	2020	2021	2022	2023	2024	
10	6	5	37	9	23	5	7	34	42	52	50	16	10	14	4	314
69	18	4	98	15	37	20	15	65	125	171	184	41	28	29	8	858
131	113	132	291	137	143	151	153	334	356	1282	546	178	126	131	50	4123
410	3	1	6	5	25	17	15	33	25	57	53	43	23	41	61	408
167	0	1	3	1	3	18	21	21	18	28	38	41	65	111	96	465
All STs	232	267	1507	442	667	395	478	1150	1615	3659	3112	910	665	790	453	16342

Supplementary Table 3. Pairwise Fisher's exact test (two-sided) for the proportion of UTI isolates from different sequence types. P-values adjusted by the Bonferroni correction to account for Family-wise error rate. P-values lower than 0.05 were considered significant. The ST167 pairwise comparisons are shown.

ST # one	ST # two	p_value	adjusted_p
10	167	3.07E-47	5.84E-45
167	131	3.32E-02	1.00E+00
167	410	6.42E-01	1.00E+00
167	69	1.46E-10	2.77E-08
167	405	5.94E-01	1.00E+00
167	361	2.71E-01	1.00E+00
167	58	3.68E-14	7.00E-12
167	38	2.57E-07	4.89E-05
167	162	9.25E-08	1.76E-05
167	648	1.21E-01	1.00E+00
167	504	9.32E-09	1.77E-06
167	297	2.89E-06	5.49E-04
167	48	9.20E-07	1.75E-04
167	1193	2.28E-01	1.00E+00
167	88	3.46E-05	6.58E-03
167	73	1.41E-06	2.69E-04
167	349	2.20E-04	4.18E-02
167	3580	2.34E-05	4.45E-03
167	95	9.15E-06	1.74E-03

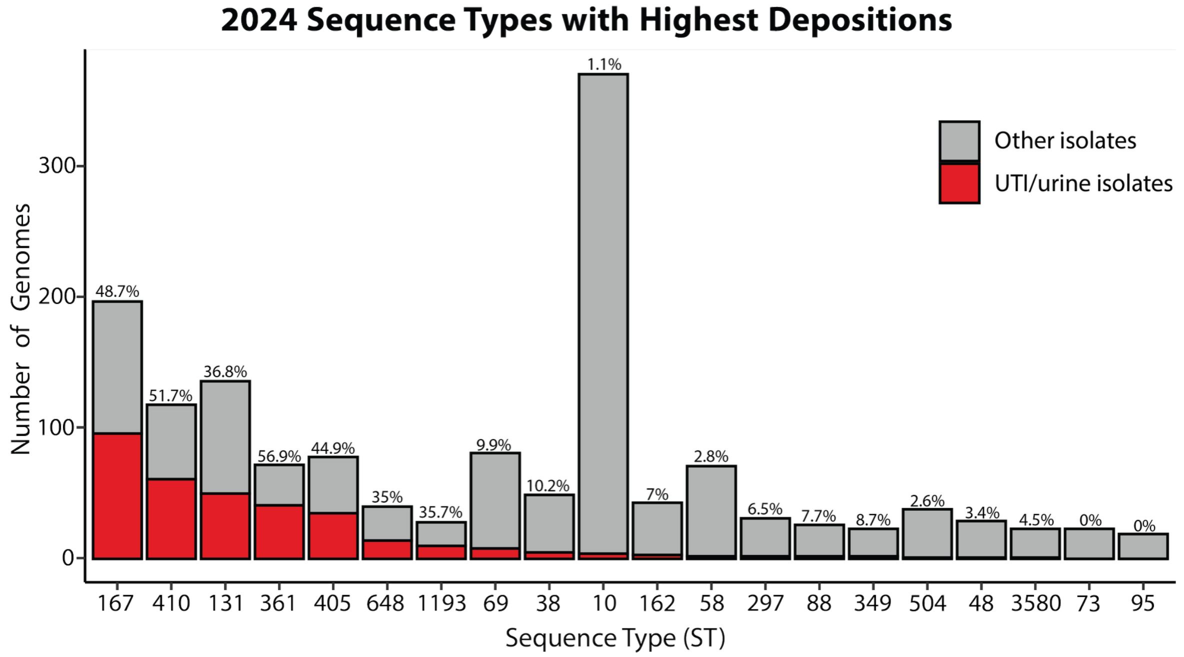

Supplementary Figure 1. Bar graph showing the 20 most deposited STs associated with human UTI/urine isolation in the EnteroBase[1] dataset in 2024. Each bar represents the number of isolates of an ST associated with an isolation source, red represents the number of isolates associated with human UTI/urine, and grey represents the number of isolates associated with all other sources. The percentage above each column represents the number of UTI/urine isolates divided by the total number of genomes in the respective ST. A pairwise Fisher's exact test for the proportion of UTI isolates from different sequence types did not lead to statistical significance (p value > 0.05) for ST167 compared to ST131, ST410, ST405, ST361, ST648 and ST1193 after correction of multiple comparisons by the Bonferroni correction. ST131 and ST1193 (phylogroup B2), ST410 (phylogroup C), ST405 (phylogroup D), and ST648 (phylogroup F) are major antibiotic resistant UPEC clones associated with high rates of UTI and bloodstream infection; less is known about ST361. In contrast, the increase in the number of ST167 sequenced isolates was statistically significant (p value < 0.05) compared to ST69, ST38, ST10, ST162, ST58, ST297, ST88, ST349, ST504, ST48, ST3580, ST73 and ST95 (Supplementary Table 1). Among these STs, ST69, ST73 and ST95 are major UPEC clones.

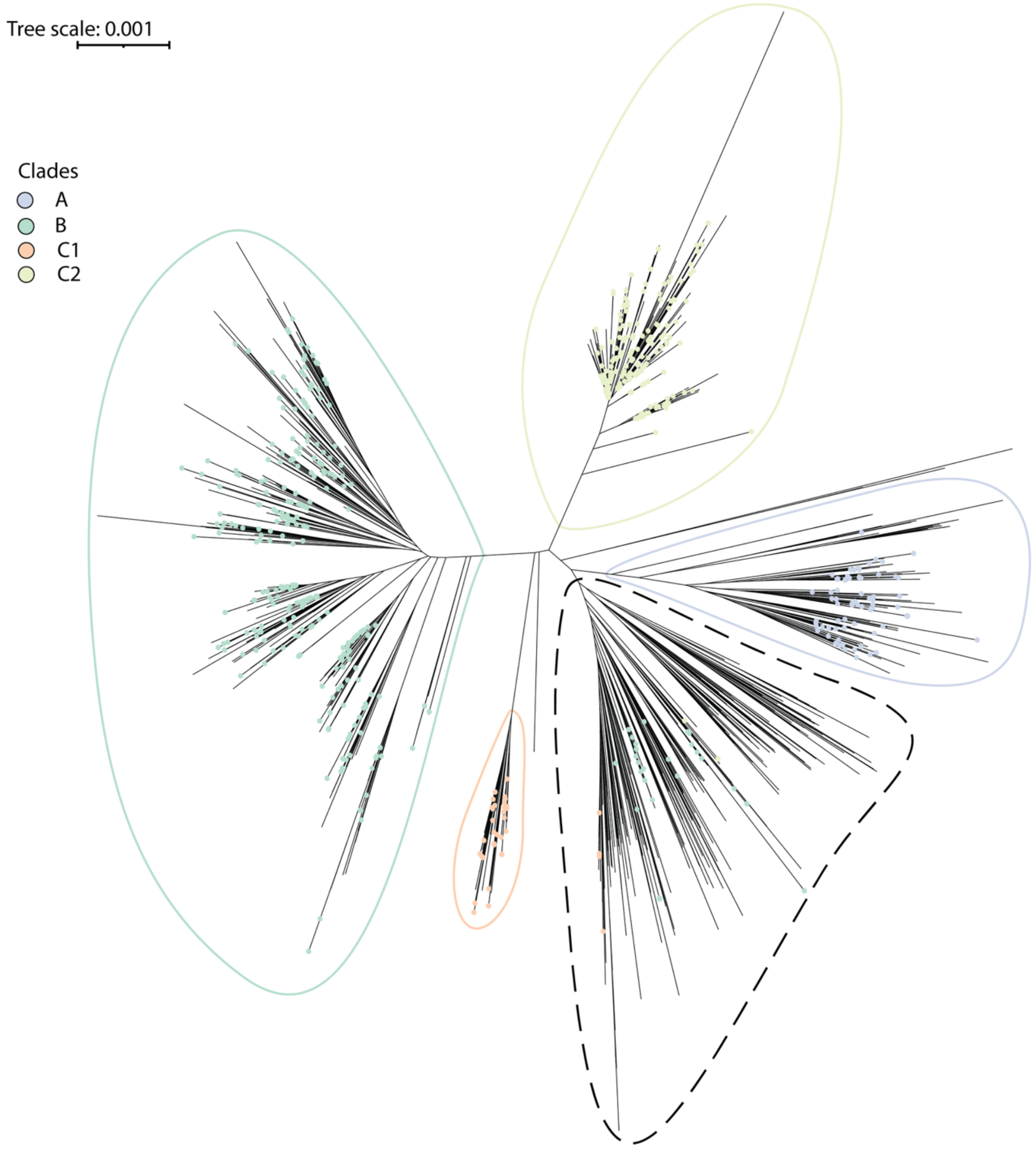

Supplementary Figure 2. A Mashtree[2] of the 2,066 ST167 genomes. The five putative clades identified by initial phylogenetic analysis are encompassed by circles, colours correspond to confirmed clades or subclades in the maximum likelihood tree, with the dotted line defining the unresolved clade identified in the Mashtree[2] but not the maximum likelihood tree. The genomes from the maximum likelihood tree of ST167 were depicted with their clades shown by tip point colours. The tree scale is representative of mash distance.

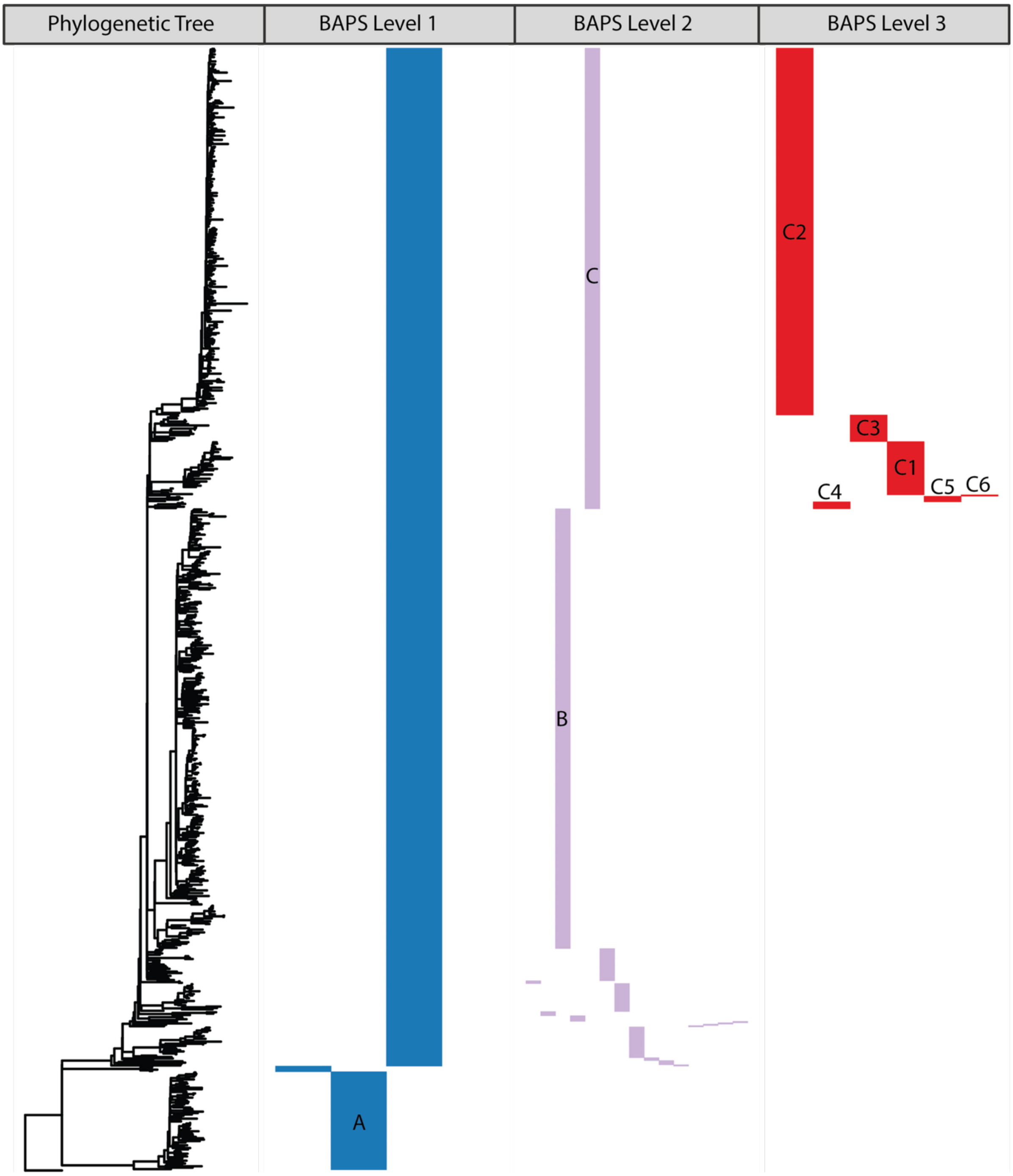

Supplementary Figure 3. Bayesian analysis of population structure by fastbaps[3] at three sequential levels. Clade A was defined at BAPS level 1 (blue), clade B and C at BAPS level 2 (purple) and the clade C subclades at BAPS level 3 (red).

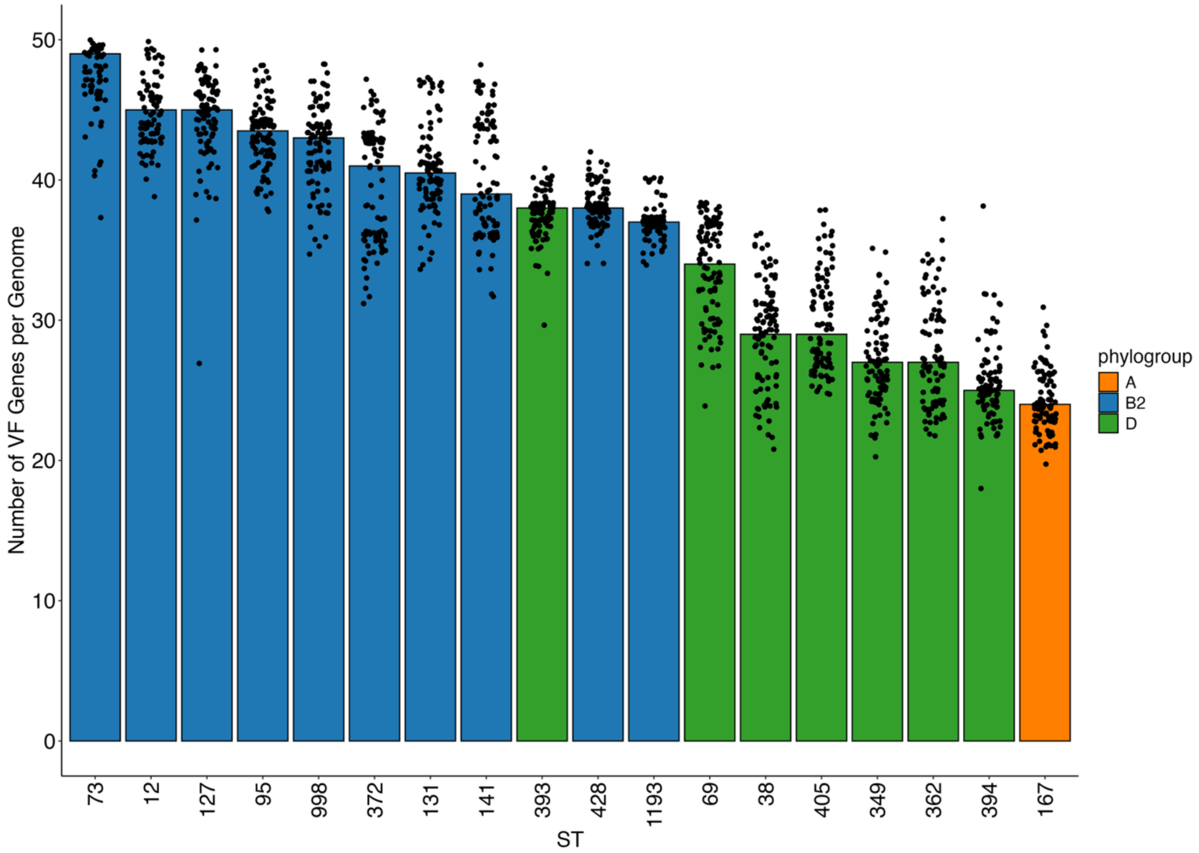

Supplementary Figure 4. Bar graph depicting the median number of virulence genes for 100 randomly selected genomes from selected UPEC STs sourced from EnteroBase. The genomes belong to the 100ST dataset previously published[4]. STs are coloured by phylogroups as indicated. A virulence factor score was determined by summing the number of virulence genes identified. A list of the virulence genes is provided in Supplementary Data 4.

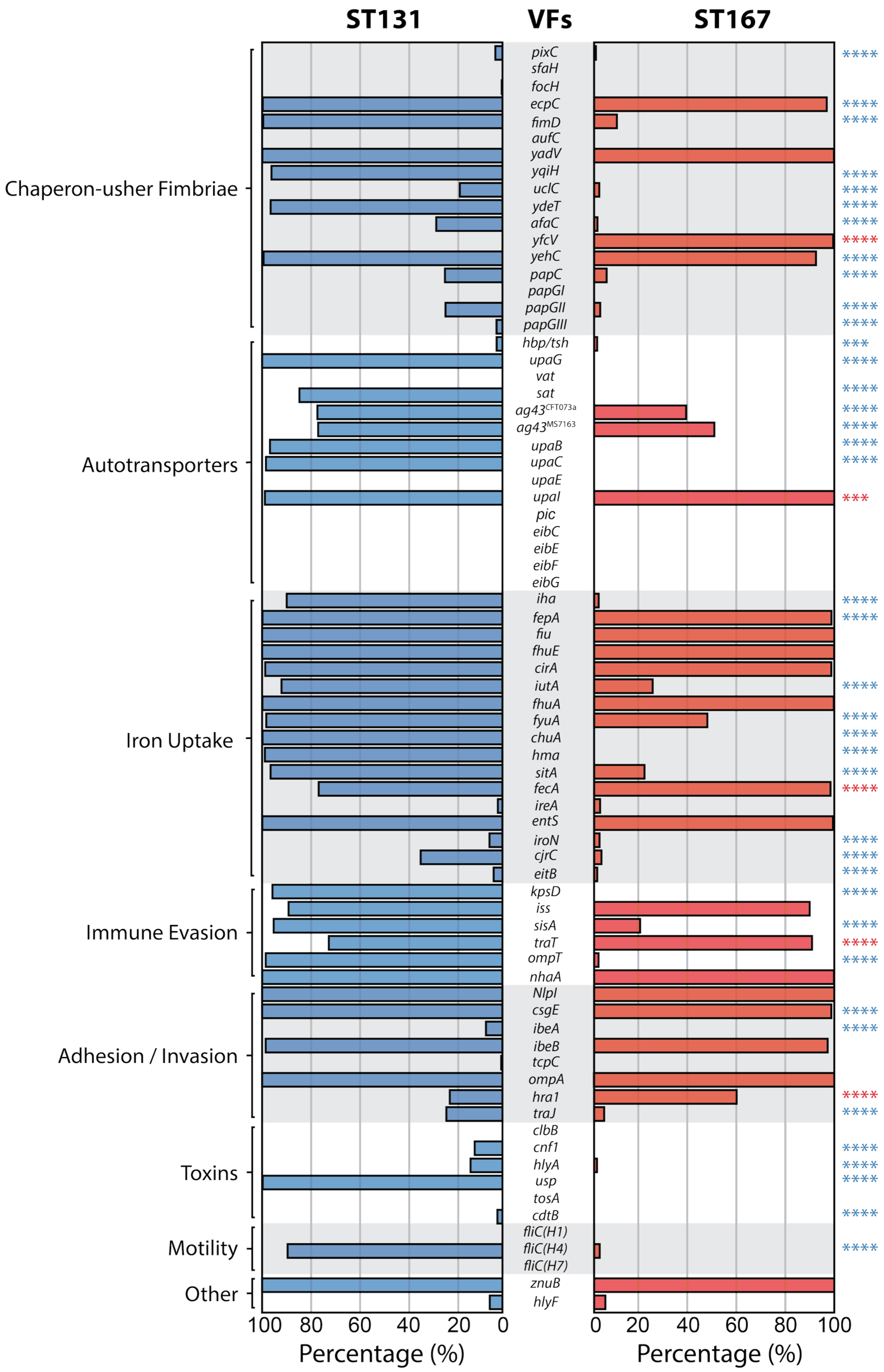
ST131
VFs
ST167
Chaperon-usher Fimbriae
Autotransporters
Iron Uptake
Immune Evasion
Adhesion / Invasion
Toxins
Motility
Other
pixC
sfaH
focH
ecpC
fimD
aufC
yadV
yqiH
uclC
ydeT
afaC
yfcV
yehC
papC
papGI
papGII
papGIII
hbp/tsh
upaG
vat
sat
ag43CFT073a
ag43MS7163
upaB
upaC
upaE
upaI
pic
eibC
eibE
eibF
eibG
iha
fepA
fiu
fhuE
cirA
iutA
fhuA
fyuA
chuA
hma
sitA
fecA
ireA
entS
iroN
cjrC
eitB
kpsD
iss
sisA
traT
ompT
nhaA
NlpI
csgE
ibeA
ibeB
tcpC
ompA
hra1
traJ
clbB
cnf1
hlyA
usp
tosA
cdtB
fliC(H1)
fliC(H4)
fliC(H7)
znuB
hlyF
100 80 60 40 20 0
0 20 40 60 80 100
Percentage (%)
Percentage (%)

Supplementary Figure 5. Comparative carriage of virulence genes in ST131 and ST167. The 2,066 available ST167 genomes and a previously published ST131 dataset (3,993 genomes) were screened by ABRicate to determine the carriage of virulence factor marker genes. The analysis used a curated dataset of established UPEC virulence factor marker genes (Supplementary Data 4). To assess a statistically significant difference in proportion of carriage, a Chi-squared test with Yates correction. The Bonferroni correction was performed to adjust for multiple testings, with significance levels indicated by asterisks: *** $p<0.001$, **** $p<0.0001$, after Bonferroni correction. Colouring of asterisks red indicates higher proportion in ST167 and colouring in blue indicates higher proportion in ST131.

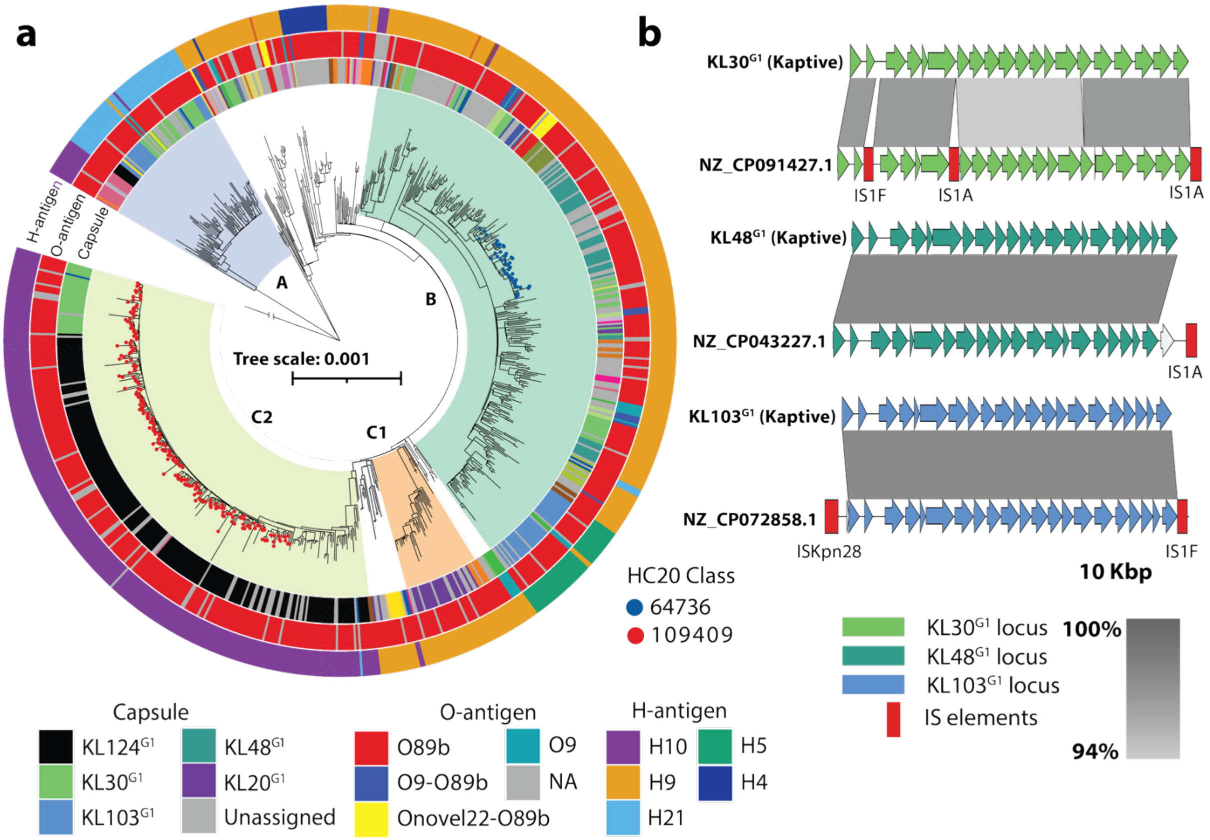

Supplementary Figure 6. a, Maximum likelihood phylogenetic tree of ST167 isolates with annotated serotype information displayed as concentric data rings. **b,** Sequence comparison of *Klebsiella* capsule loci references from Kaptive 2.0[5] and completely sequenced ST167 genomes retrieved from RefSeq. Grey shading between sequences indicates nucleotide similarity between sequences as determined by Easyfig[6]. IS elements were annotated with ISfinder[7].

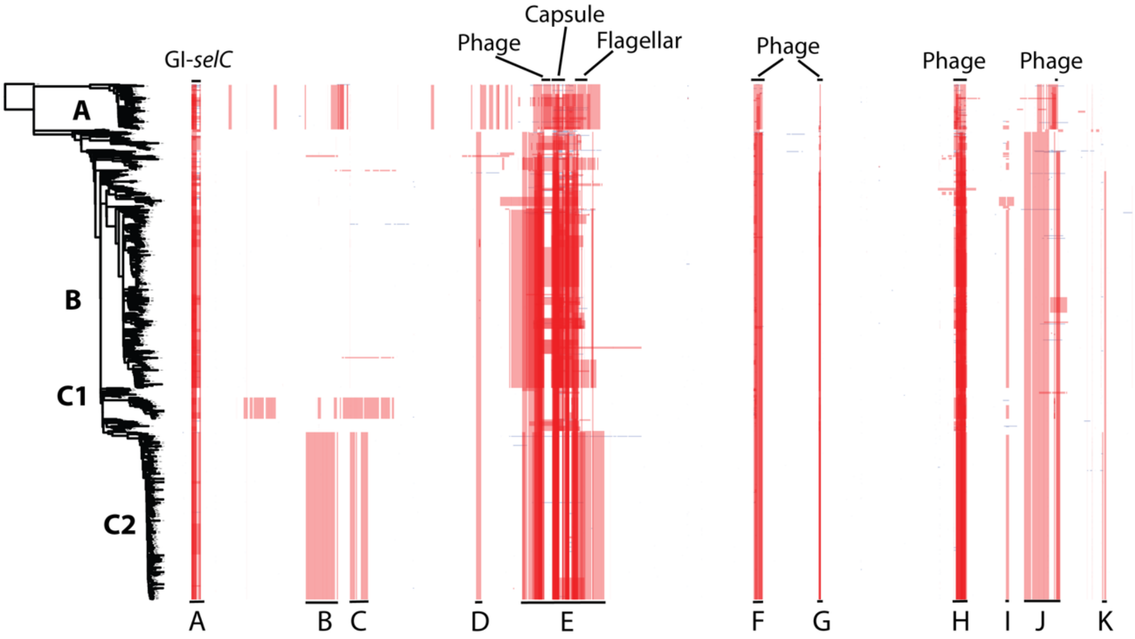

Supplementary Figure 7. Visualisation of the ST167 phylogenetic tree aligned to Gubbins[8] predicted recombination regions throughout the aligned pseudo-genome of ST167. Red blocks indicate predicted recombination events, darker shades of red represent overlapping predicted recombinant regions. The image was visualised in Phandango[9]. Phage regions were identified by PHASTEST[10].

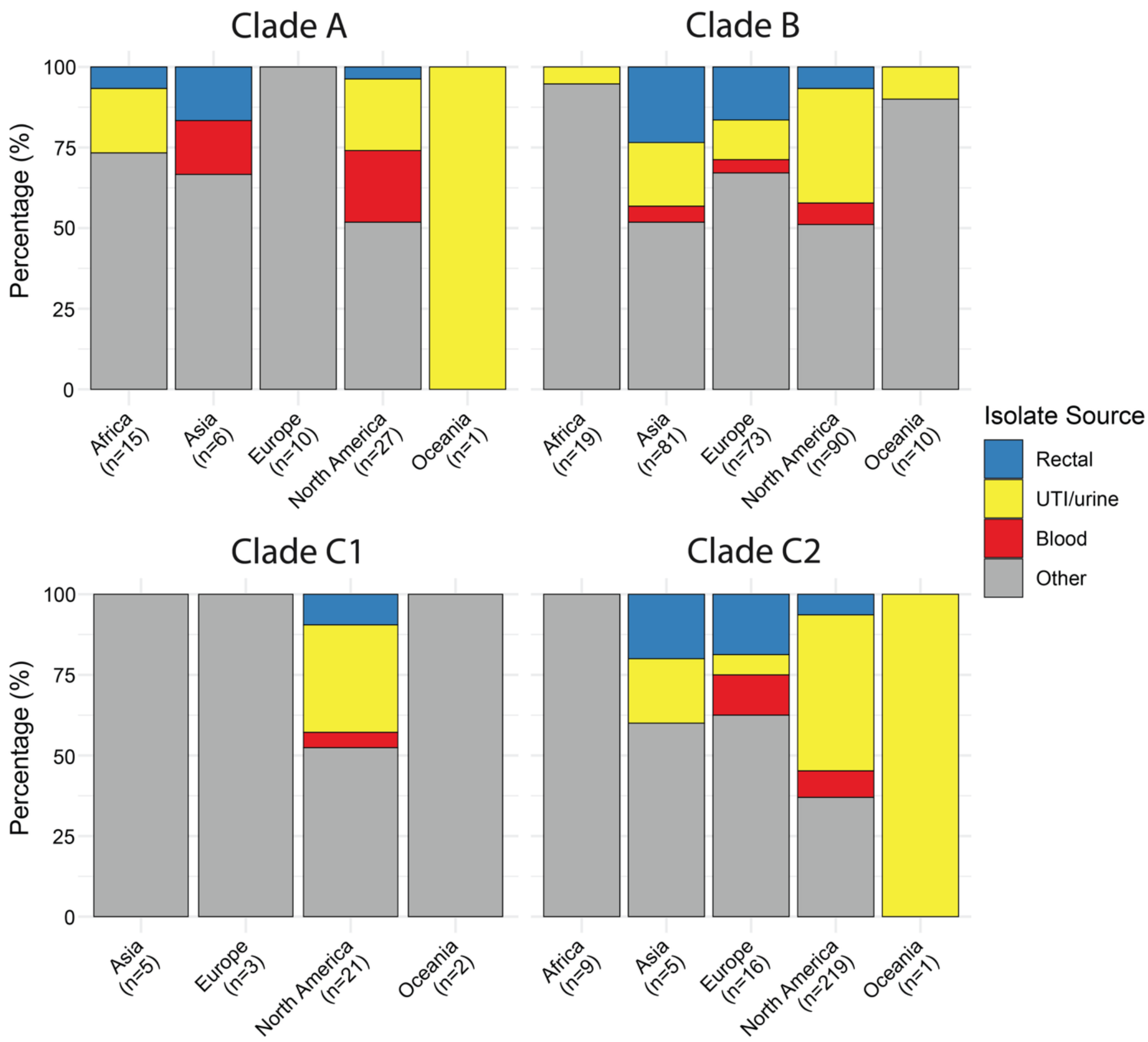

Supplementary Fig 8. Column graphs describing the proportion of isolates from different sources for each phylogenetic clade and geographical region. The total number of isolates sourced from each geographical region (n) is noted in brackets. Isolate sources are colour coded according to the legend.

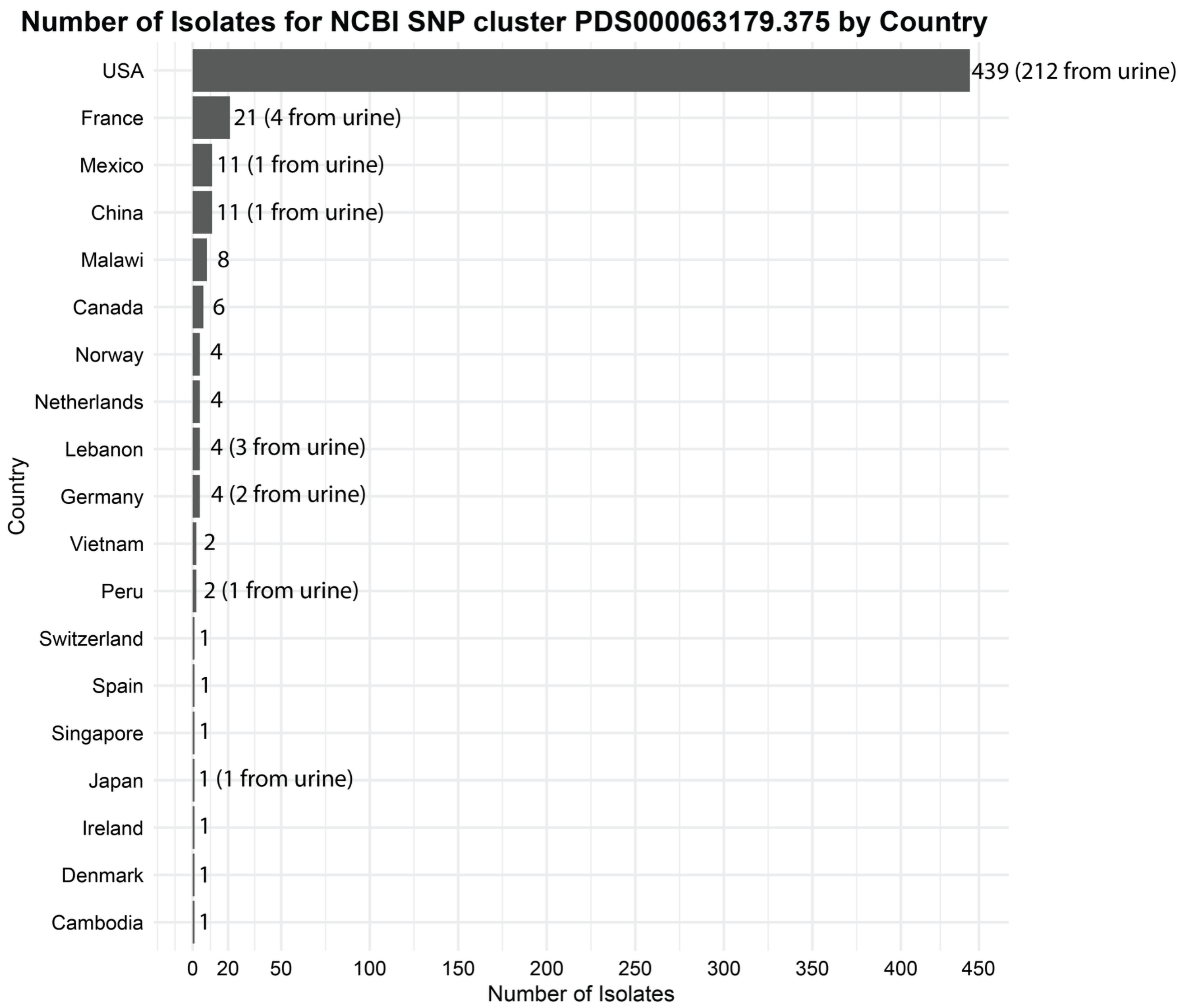

Supplementary Fig 9. Bar graph depicting the number of isolates from NCBI pathogen detection SNP cluster PDS000063179.375 per country (total isolates = 528). Metadata for genomes can be found at NCBI pathogen detection Isolates were clustered using whole genome MLST (wgMLST) with a clustering threshold of <25 allelic differences.

References

1 Zhou, Z., Alikhan, N. F., Mohamed, K., Fan, Y. & Achtman, M. The EnteroBase user's guide, with case studies on *Salmonella* transmissions, *Yersinia pestis* phylogeny, and *Escherichia* core genomic diversity. *Genome Res* **30**, 138-152 (2020). https://doi.org/10.1101/gr.251678.119

2 Katz, L. S. *et al.* Mashtree: a rapid comparison of whole genome sequence files. *J Open Source Softw* **4** (2019). https://doi.org/10.21105/joss.01762

3 Tonkin-Hill, G., Lees, J. A., Bentley, S. D., Frost, S. D. W. & Corander, J. Fast hierarchical Bayesian analysis of population structure. *Nucleic Acids Res* **47**, 5539-5549 (2019). https://doi.org/10.1093/nar/gkz361

4 Phan, M.-D. *et al.* Plasmid-Mediated Ciprofloxacin Resistance Imparts a Selective Advantage on *Escherichia coli* ST131. *Antimicrobial Agents and Chemotherapy* **66**, e02146-02121 (2022). https://doi.org/doi:10.1128/AAC.02146-21

5 Lam, M. M. C., Wick, R. R., Judd, L. M., Holt, K. E. & Wyres, K. L. Kaptive 2.0: updated capsule and lipopolysaccharide locus typing for the *Klebsiella pneumoniae* species complex. *Microbial Genomics* **8** (2022). https://doi.org/https://doi.org/10.1099/mgen.0.000800

6 Sullivan, M. J., Petty, N. K. & Beatson, S. A. Easyfig: a genome comparison visualizer. *Bioinformatics* **27**, 1009-1010 (2011). https://doi.org/10.1093/bioinformatics/btr039

7 Siguier, P., Perochon, J., Lestrade, L., Mahillon, J. & Chandler, M. ISfinder: the reference centre for bacterial insertion sequences. *Nucleic Acids Res* **34**, D32-36 (2006). https://doi.org/10.1093/nar/gkj014

8 Croucher, N. J. *et al.* Rapid phylogenetic analysis of large samples of recombinant bacterial whole genome sequences using Gubbins. *Nucleic Acids Research* **43**, e15-e15 (2014). https://doi.org/10.1093/nar/gku1196

9 Hadfield, J. *et al.* Phandango: an interactive viewer for bacterial population genomics. *Bioinformatics* **34**, 292-293 (2017). https://doi.org/10.1093/bioinformatics/btx610

10 Wishart, D. S. *et al.* PHASTEST: faster than PHASTER, better than PHAST. *Nucleic Acids Res* **51**, W443-w450 (2023). https://doi.org/10.1093/nar/gkad382
