# Supplementary material for: Emergence of a carbapenem-resistant atypical uropathogenic Escherichia coli clone as an increasing cause of urinary tract infection
Source: Nat Commun. 2025 Sep 2;16:8200. doi: 10.1038/s41467-025-63477-0 (PMC12405470; doi:10.1038/s41467-025-63477-0)
Supplement: Supplementary file 2 — Description of Additional Supplementary Files [file 41467_2025_63477_MOESM2_ESM.pdf]

Description of additional Supplementary Data files

Title: Supp. Data 1
Description: Phylogenetic clusters identified by FastBAPS

Title: Supp. Data 2
Description: Genome Metadata for ST167 isolates

Title: Supp. Data 3
Description Antibiotic susceptibility testing

Title: Supp. Data 4
Description: Virulence factor database

Title: Supp. Data 5
Description Recombination regions identified

Title: Supp. Data 6
Description BEAST analysis

Title: Supp. Data 7
Description: Carbapenem resistant E. coli meta-analysis

Title: Supp. Data 8
Information on contributing sequencing projects in EnteroBase dataset
