# Supplementary material for: Emergence of a carbapenem-resistant atypical uropathogenic Escherichia coli clone as an increasing cause of urinary tract infection
Source: Nat Commun. 2025 Sep 2;16:8200. doi: 10.1038/s41467-025-63477-0 (PMC12405470; doi:10.1038/s41467-025-63477-0)
Supplement: Supplementary file 11 — Reporting Summary [file 41467_2025_63477_MOESM11_ESM.pdf]

nature portfolio

Corresponding author(s): Mark Schembri, Brian Forde

Last updated by author(s): Aug 3, 2025

Reporting Summary

Nature Portfolio wishes to improve the reproducibility of the work that we publish. This form provides structure for consistency and transparency in reporting. For further information on Nature Portfolio policies, see our Editorial Policies and the Editorial Policy Checklist.

Statistics

For all statistical analyses, confirm that the following items are present in the figure legend, table legend, main text, or Methods section.

n/a	Confirmed	
☐	☒	The exact sample size (*n*) for each experimental group/condition, given as a discrete number and unit of measurement
☒	☐	A statement on whether measurements were taken from distinct samples or whether the same sample was measured repeatedly
☐	☒	The statistical test(s) used AND whether they are one- or two-sided<br>*Only common tests should be described solely by name; describe more complex techniques in the Methods section.*
☒	☐	A description of all covariates tested
☐	☒	A description of any assumptions or corrections, such as tests of normality and adjustment for multiple comparisons
☒	☐	A full description of the statistical parameters including central tendency (e.g. means) or other basic estimates (e.g. regression coefficient) AND variation (e.g. standard deviation) or associated estimates of uncertainty (e.g. confidence intervals)
☒	☐	For null hypothesis testing, the test statistic (e.g. *F*, *t*, *r*) with confidence intervals, effect sizes, degrees of freedom and *P* value noted<br>*Give P values as exact values whenever suitable.*
☐	☒	For Bayesian analysis, information on the choice of priors and Markov chain Monte Carlo settings
☐	☒	For hierarchical and complex designs, identification of the appropriate level for tests and full reporting of outcomes
☒	☐	Estimates of effect sizes (e.g. Cohen's *d*, Pearson's *r*), indicating how they were calculated

Our web collection on statistics for biologists contains articles on many of the points above.

Software and code

Policy information about availability of computer code

Data collection	Genomic metadata to determine the prevalence of STs was retrieved from Enterobase (https://enterobase.warwick.ac.uk). ST167 genome assemblies were downloaded from Enterobase (https://enterobase.warwick.ac.uk) based off seven gene MLST.
Data analysis	Data analyses was performed using the following tools:<br>QUAST version 5, available at: https://github.com/ablab/quast<br>Mashtree version 1.4.6, available at: https://github.com/lskatz/mashtree<br>Prokka version 1.13, available at: https://github.com/tseemann/prokka<br>Snippy version 4.6.0, available at: https://github.com/tseemann/snippy<br>Gubbins version 3.2.1, available at: https://github.com/nickjcroucher/gubbins<br>IQtree version 2.1.2, available at: https://github.com/iqtree/iqtree2<br>AMRfinderplus version 3.11.20, available at: https://github.com/ncbi/amr<br>ABRicate version 1.0.1, available at: https://github.com/tseemann/abricate<br>Easyfig version 2.2.2, available at: https://mjsull.github.io/Easyfig/<br>Kaptive version 2.0, available at: https://github.com/klebgenomics/Kaptive<br>PHASTEST, available at: https://phastest.ca/<br>BEAST, version 2.6.7, available at: https://github.com/CompEvol/beast2/releases/tag/v2.6.7<br>TempEST version 1.5.3, available at https://github.com/beast-dev/Tempest<br>Tracer version 1.7.2, available at https://github.com/beast-dev/tracer<br><br>R packages include:

Fastbaps version 1.0.8, available at: https://github.com/gtonkinhill/fastbaps
The R Stats Package version 4.1.3, available at https://cran.r-project.org/bin/macosx/ (Base R)

For manuscripts utilizing custom algorithms or software that are central to the research but not yet described in published literature, software must be made available to editors and reviewers. We strongly encourage code deposition in a community repository (e.g. GitHub). See the Nature Portfolio guidelines for submitting code & software for further information.

Data

Policy information about availability of data

All manuscripts must include a data availability statement. This statement should provide the following information, where applicable:

- Accession codes, unique identifiers, or web links for publicly available datasets
- A description of any restrictions on data availability
- For clinical datasets or third party data, please ensure that the statement adheres to our policy

All data generated in this work is presented in the manuscript. Supplementary data 1 contains phylogenetic clusters identified by FastBAPS. Supplementary data 2 contains the accession numbers, metadata, serotype and AMR genes of all ST167 genomes. Supplementary data 3 contains the antibiotic susceptibility data of the ST167 isolates. Supplementary data 4 contains the information pertaining to the virulence factor database used. Supplementary data 5 contains all of the genes found in recombination regions. Supplementary data 6 contains analysis of BEAST data. Supplementary data 7 contains the data for the carbapenem resistant E. coli meta-analysis. Supplementary data 8 contains information on the contributing sequencing projects. Enterobase data pertaining to number of genomes submitted each year per ST, prevalence in UTI dataset and statistics are found in Supplementary Table 1, 2 and 3.

Human research participants

Policy information about studies involving human research participants and Sex and Gender in Research.

Reporting on sex and gender	No human research participants were involved in this study.
Population characteristics	No human research participants were involved in this study.
Recruitment	No human research participants were involved in this study.
Ethics oversight	No human research participants were involved in this study.

Note that full information on the approval of the study protocol must also be provided in the manuscript.

Field-specific reporting

Please select the one below that is the best fit for your research. If you are not sure, read the appropriate sections before making your selection.

[x] Life sciences [ ] Behavioural & social sciences [ ] Ecological, evolutionary & environmental sciences

For a reference copy of the document with all sections, see nature.com/documents/nr-reporting-summary-flat.pdf

Life sciences study design

All studies must disclose on these points even when the disclosure is negative.

Sample size	Sample sizes were not predetermined based on statistical methods, but were chosen according to the standards in the field and computational limitations.
Data exclusions	No data were excluded from the analyses.
Replication	Phylogenetic analyses were validated with known software and contrasted with differing methodology.<br><br>Bayesian analyses were tested with multiple replicates to ensure convergence of data.
Randomization	No randomization was necessary as experiments were performed with appropriate controls. Randomization is not generally used in this field.
Blinding	Investigators were not blinded. Blinding during analysis was not necessary because the results are quantitative and did not require subjective judgment or interpretation. Blinding is not typically used in the field.

Reporting for specific materials, systems and methods

We require information from authors about some types of materials, experimental systems and methods used in many studies. Here, indicate whether each material, system or method listed is relevant to your study. If you are not sure if a list item applies to your research, read the appropriate section before selecting a response.

Materials & experimental systems

n/a	Involved in the study	
x		Antibodies
x		Eukaryotic cell lines
x		Palaeontology and archaeology
	x	Animals and other organisms
x		Clinical data
x		Dual use research of concern

Methods

n/a	Involved in the study	
x		ChIP-seq
x		Flow cytometry
x		MRI-based neuroimaging

Animals and other research organisms

Policy information about studies involving animals; ARRIVE guidelines recommended for reporting animal research, and Sex and Gender in Research

Laboratory animals	Female C57BL/6 mice 8 to 10 weeks old were used.
Wild animals	The study did not involve wild animals
Reporting on sex	Female adult mice are primarily used in mouse UTI models as they are represent the gender bias in human UTIs.
Field-collected samples	The study did not involve collection of field samples.
Ethics oversight	Mouse infection experiments were approved by the University of Queensland Animal Ethics Committee (AEC approval number SCMB/259/19).

Note that full information on the approval of the study protocol must also be provided in the manuscript.
